# Global Prevalence of Macroprolactinemia among Patients with Hyperprolactinemia: A Systematic Review and Meta-Analysis

**DOI:** 10.3390/ijerph17218199

**Published:** 2020-11-06

**Authors:** Noor Azlin Azraini Che Soh, Najib Majdi Yaacob, Julia Omar, Aniza Mohammed Jelani, Noorazliyana Shafii, Tuan Salwani Tuan Ismail, Wan Norlina Wan Azman, Anis Kausar Ghazali

**Affiliations:** 1 Department of Chemical Pathology, School of Medical Sciences, Universiti Sains Malaysia, Health Campus, Kubang Kerian 16150, Kelantan, Malaysia; noorazlin79@usm.my (N.A.A.C.S.); juliakb@usm.my (J.O.); anizamj@usm.my (A.M.J.); noorazliyana@usm.my (N.S.); tusti@usm.my (T.S.T.I.); dr_wannorlina@usm.my (W.N.W.A.); 2 Unit of Biostatistics and Research Methodology, School of Medical Sciences, Universiti Sains Malaysia, Health Campus, Kubang Kerian 16150, Kelantan, Malaysia; anisyo@usm.my

**Keywords:** macroprolactin, macroprolactinemia, big-big prolactin, prolactin, hyperprolactinemia, prevalence, meta-analysis

## Abstract

Hyperprolactinemia (hPRL) often poses a diagnostic dilemma due to the presence of macroprolactin. Understanding the prevalence of macroprolactinemia (mPRL) has an important implication in managing patients with hPRL. The primary aim of this study was to determine the prevalence of mPRL globally and to explore selected factors influencing the prevalence estimate. Studies with original data related to the prevalence of mPRL among patients with hPRL from inception to March 2020 were identified, and a random effects meta-analysis was performed. Of the 3770 records identified, 67 eligible studies from 27 countries were included. The overall global prevalence estimate was 18.9% (95% CI: 15.8%, 22.1%) with a substantial statistical heterogeneity (I^2^ = 95.7%). The highest random effects pooled prevalence was observed in the African region (30.3%), followed by Region of the Americas (29.1%), European (17.5%), Eastern Mediterranean (13.9%), South-East Asian (12.7%), and Western Pacific Region (12.6%). Lower prevalence was observed in studies involving both sexes as compared to studies involving only female participants (17.1% vs. 25.4%) and in more recent studies (16.4%, 20.4%, and 26.5% in studies conducted after 2009, between 2000 and 2009, and before 2000, respectively). The prevalence estimate does not vary according to the age group of study participants, sample size, and types of polyethylene glycol (PEG) used for detection of macroprolactin (PEG 6000 or PEG 8000). With macroprolactin causing nearly one-fifth of hPRL cases, screening for mPRL should be made a routine before an investigation of other causes of hPRL.

## 1. Introduction

Prolactin (PRL) is a hormone secreted by lactotroph cells within the adenohypophysis. PRL is synthesized as a prehormone with a molecular weight of 26 kDa [1]. PRL exists in different forms in human serum. The predominant form is monomeric PRL (little PRL) with a molecular mass of 23 kDa. The other forms include dimeric PRL (big PRL) with a molecular mass of 48–56 kDa, and another form is polymeric PRL, also known as macroprolactin (big-big PRL), with molecular mass >150 kDa. In the normal sera, monomeric PRL accounts for 80–95% of the total PRL, dimeric PRL makes up <10%, and macroprolactin accounts for a small amount of less than 1% of the total PRL [2].

The monomeric PRL is known to be biologically and immunologically active, and when it is in excess, it will cause hyperprolactinemia (hPRL) [2]. When the serum of a patient with hPRL contains mostly macroprolactin, the condition is termed macroprolactinemia (mPRL) [3,4]. In up to 90% of cases, macroprolactin is composed of a complex formed by an IgG and monomeric PRL [4,5,6]. Macroprolactin has a prolonged clearance rate like that of immunoglobulins [7].

Macroprolactin is confined to the vascular system and has limited access to the PRL receptor of target organs owing to limited bioactivity in vivo resulting in asymptomatic hPRL [8,9]. In true hPRL, the common clinical syndromes include galactorrhoea, oligomenorrhoea, or amenorrhoea, and infertility in women and reduced libido, oligospermia or impotence or both, and galactorrhoea in men but not in mPRL. However, it causes diagnostic confusion when it is coincidentally associated with hyperprolactinemic syndrome’s non-specific symptoms. In these circumstances, the symptoms may be mistakenly attributed to true hPRL [10,11]. Therefore, the differentiation between true hPRL and mPRL cannot be made solely based on clinical symptoms. Although macroprolactin is generally biologically inactive, it can be measured by almost all immunoassays for PRL [7,12,13,14]. This may lead to misdiagnosis and unnecessary medical and surgical intervention [15,16] or delayed diagnosis, and inappropriate treatment [17,18].

Screening of hPRL sera for the presence of misleading concentrations of mPRL must be included in the routine investigation of all hyperprolactinemic patients. The reference method for the determination of macroprolactin is gel filtration chromatography (GFC), which allows quantitation of all high molecular mass forms of PRL and an estimate of their molecular mass. Although the GFC method is accurate and reproducible, it is expensive, labor-intensive, and time-consuming. Many alternative techniques have been described based on immunoassay of serum PRL before and after removal of macroprolactin by ultrafiltration, immunoadsorption of IgG species with protein A, protein G, or anti-human IgG and precipitation with polyethylene glycol (PEG) [19,20,21].

PEG precipitation is the best, most widely used method and recommended worldwide for detecting macroprolactin as this method is reproducible, easily performed, and effective. One limitation of PEG precipitation has been reported in which the presence of PEG in the sample can interfere with some PRL immunoassay procedures [22]. To overcome this problem, each laboratory must establish its reference intervals derived from PEG-treated sera of healthy individuals [11,15].

Although many studies have reported the prevalence of mPRL among hPRL using various immunoassay analyzers, different methods of detection for mPRL, different cut-off PRL levels for the screening of mPRL, and various cut-off recovery post-PEG, a systematic literature review on the prevalence of mPRL had not been performed to date. The primary objectives of this study were to conduct a systematic literature review and meta-analysis on the prevalence of mPRL, summarize the findings of these studies, and explore selected factors that may influence prevalence estimates.

## 2. Materials and Methods

### 2.1. Protocol and Registration

This systematic review and meta-analysis followed the Preferred Reporting Items for Systematic Reviews and Meta-analyses (PRISMA) checklist. The protocol was registered in the PROSPERO international prospective register of systematic reviews (PROSPERO registration number: CRD42019123884).

### 2.2. Data Sources and Search Strategies

Two investigators (N.A.A.C.S. and N.M.Y.) extensively searched online international databases subscribed by our institutional library (PubMed, EMBASE, Cochrane Library Database, SAGE, Scopus, EBSCO Academic Search Complete, EBSCO PsycINFO, ProQuest, Elsevier, ScienceDirect, Google Scholar, and Emerald Insight) from inception to 30 March 2020. The search terms were MeSH terms and text words linked to mPRL and hPRL using a combination of the following search terms: “polymeric prolactin”, “macroprolactin”, “macroprolactinemia”, “macroprolactinaemia”, “big big prolactin”, “BBPRL”, “big prolactin”, “BPRL”, “hyperprolactinemia”, “hyperprolactinaemia”, “elevated prolactin”, “excess prolactin”, “high prolactin”. The search strategy was tested in two databases (PubMed, Elsevier ScienceDirect) and was further refined based on its ability to retrieve known relevant studies according to each database. Forward and backward reference chaining of included studies were carried out in which the reference lists from the included papers were searched to identify other relevant information. A systematic literature search of multiple databases using search terms as listed above was conducted to search for articles published in peer-reviewed literature, clinical trial registries, conference proceedings, and gray literature. To maximize sensitivity rather than the specificity of the literature search, we did not include “prevalence”, “incidence”, “proportion”, or “frequency” as the search term.

### 2.3. Study Eligibility

Two investigators (N.A.A.C.S. and N.M.Y.) independently screened all titles and abstracts from the initial search results and full-text articles identified from the first-stage screening (titles and abstract). Studies that reported primary data on the prevalence of mPRL from inception to 30 March 2020 were included. Searches were conducted in English, and publications in all languages were considered. Any observational (cross-sectional, cohort, longitudinal) studies were eligible for inclusion if the study reported the target population of interest (hPRL patients regardless of cause) and on study outcomes (prevalence or frequency of mPRL among hPRL patients). Experimental (randomized, non-randomized) trials, case-control studies, ecological studies, case reports, studies that did not involve human participants (animal, in vitro studies), book chapters, narrative reviews, and protocol studies were excluded.

### 2.4. Data Extraction

Search results from each database were downloaded in a standardized tag format developed by Research Information Systems (.ris) or NBIB format (.nbib). In databases that do not allow all search results to be downloaded at once (e.g., Google Scholar, EMBASE), search results were downloaded in partitions and later merged in Microsoft Windows command prompt (cmd) using this command: “copy *.ris mergefile.ris”. The search results were then imported into Zotero software to remove duplicates. After removing duplicates, the search result was exported as Microsoft Excel.csv format and later converted to .xlsx format.

Preliminary screening of titles and abstracts was conducted by two investigators (N.A.A.C.S. and N.M.Y.) to identify potential articles of interest. The full text of potentially eligible studies was retrieved and re-assessed for inclusion/exclusion criteria. Assessment of eligibility was made in duplication and independently to avoid bias in study selection. The degree of change-adjusted agreement between the two review authors was noted and statistically assessed by Kappa statistics. Conflicts in study identification were resolved by discussion and in conjunction with a third investigator (J.O.) to obtain 100% agreement with the final decision. A detailed assessment of why studies were excluded after the full-text review was prepared.

After study identification, data from included studies were abstracted by two investigators (N.A.A.C.S. and N.M.Y.) using a standardized pre-design and pre-piloted electronic data abstraction form in Microsoft Excel format to assess study quality and for evidence synthesis. Data abstractions were conducted independently to minimize the risk of errors. The information abstracted included: author’s name, publication year, country, region, study design, study population, operational definition of hPRL, diagnostic test for hPRL, diagnostic test for mPRL, the cut-off point for the diagnostic test used for diagnosis of mPRL, number of study participants (hPRL), and number of participants with the outcome of interest (mPRL).

When there were multiple publications of the same study, data were extracted from each publication, but only the most “complete” and up-to-date data were included. The data were analyzed following resolution of overlaps in the extracted data. The literature search and screening output were reported in a Preferred Reporting Items for Systematic Reviews and Meta-Analyses (PRISMA) study flow diagram.

### 2.5. Quality Assessment

The quality of each included study (assessment of bias) was critically and objectively appraised by two investigators (N.A.A.C.S. and N.M.Y.) independently and in duplicate, using adapted quality assessment tool for prevalence studies [23]. The tool consists of 10 items addressing three domains of bias (selection, nonresponse, measurement bias) and a summary score classifying the study as low, medium, or high risk of bias. All disagreement was resolved by discussion with the involvement of a third review author (J.O.).

### 2.6. Statistical Analyses

The qualitative synthesis omitted studies with a high risk of bias. Aggregate level data was used for data synthesis, and a summary of all the findings in the included studies was provided. A meta-analysis of the prevalence was conducted using the *metaprop* module in STATA software version 14.1 (Stata Corporation, College Station, TX, USA). A random effects meta-analysis was performed to obtain the pooled prevalence with the corresponding 95% confidence interval (95% CI) and forest plot. Confidence intervals for the pooled estimates were calculated after the Freeman–Tukey double arcsine transformation. The possibility of statistical heterogeneity among included studies was estimated by Cochran’s Q (reported with a χ^2^ and *p*-value) and the I^2^ statistic. The I^2^ statistic describes the fraction of the variability in effect that is due to heterogeneity rather than sampling error. A *p*-value of less than 0.10, rather than the conventional level of 0.05, is used to determine statistical significance of heterogeneity.

Publication bias was assessed using Egger’s test and funnel plot. Sensitivity analysis was performed by eliminating individual studies one at a time. Alteration in the pooled prevalence and the 95% CI were examined to assess the stability of the meta-analysis. Subgroup analyses were conducted according to region, sex, age group, year period when the study was published, and types of PEG used for the detection of macroprolactin. The random effect pooled prevalence estimate with the corresponding 95% CI, the within-group heterogeneity, and the between-group heterogeneity tests were reported. A *p*-value for this test of less than 0.10 indicates a statistically significant subgroup (interaction) effect. As an extension to subgroup analysis, individual variable meta-regression was conducted to investigate the effect of continuous study characteristics (sample size and year of studies) and a *p*-value of less than 0.05 was considered for statistical significance.

## 3. Results

### 3.1. Study Selection and Characteristics

**Figure 1:**
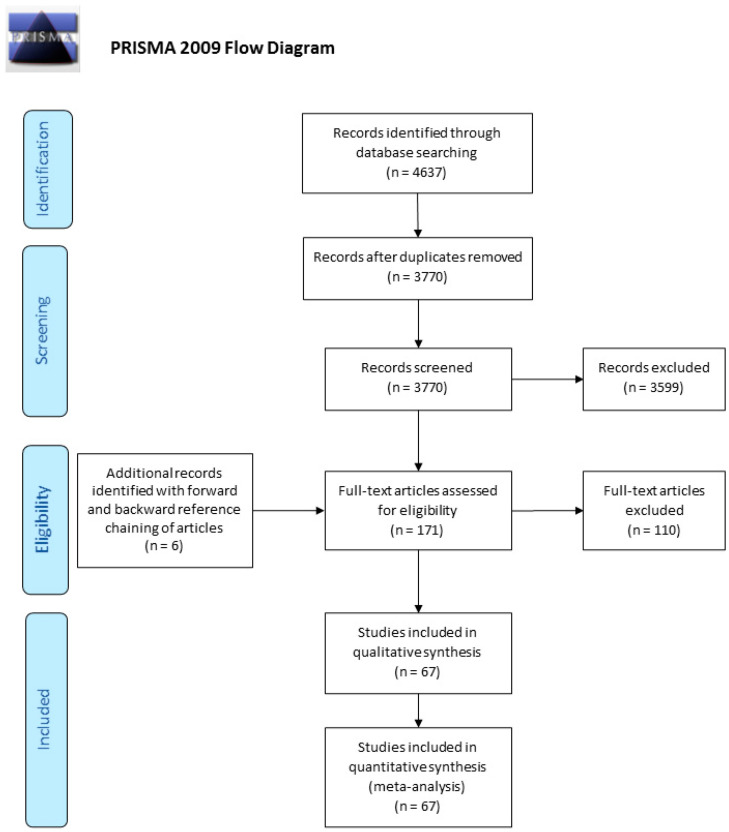
Preferred Reporting Items for Systematic Reviews and Meta-Analyses (PRISMA) flowchart.

After removal of duplicates, 3770 records were screened by their titles and abstracts from which 171 articles qualified for a full-text review. Forward and backward reference chaining of articles during full-text review identified six extra articles. In total, 177 articles were assessed for eligibility in full text, and from these, 67 studies reported on the prevalence of mPRL among patients with hPRL and fulfilled other eligibility criteria (Figure 1: Flow of information diagram). The final sample of 67 studies published between 1985 and 2019 from 27 countries was included, involving 16,951 patients with hPRL. The largest proportion of studies came from the European Region (37 studies, 55.2%) followed by Region of the Americas (14 studies, 20.9%), Western Pacific Region (7 studies, 10.4%), Eastern Mediterranean Region (4 studies, 6.0%), South-East Asia Region (3 studies, 4.5%), and African Region (2 studies, 3.0%).

Large heterogeneity between studies was observed concerning the method of hPRL and mPRL detection, as listed in Table 1. The majority of the included studies (56 studies) utilized a single method to detect hPRL, eight utilized a combination of two methods, two studies utilized a combination of three methods, and one study utilized a combination of four methods. The majority of the studies (28 studies) used Chemiluminescence Immunoassay (CLIA) as the method of detecting hPRL, and 24 studies used the Electrochemiluminescence Immunoassay (ECLIA) method. For the diagnosis of mPRL, 47 studies used a single method, 20 studies used a combination of two methods, and one study used a combination of three methods. PEG is the most used method for diagnosis of mPRL, 47 studies used PEG 6000, 6 studies used PEG 8000, and 10 studies used PEG but did not specify whether it was PEG 6000 or PEG 8000. GFC was used in 20 studies for the diagnosis of mPRL. Various recovery cut-off points were used for diagnosis of mPRL, and most of the studies used <40% PRL recovery as the cut-off.

### 3.2. Prevalence of Macroprolactinemia among Patients with Hyperprolactinemia

**Figure 2:**
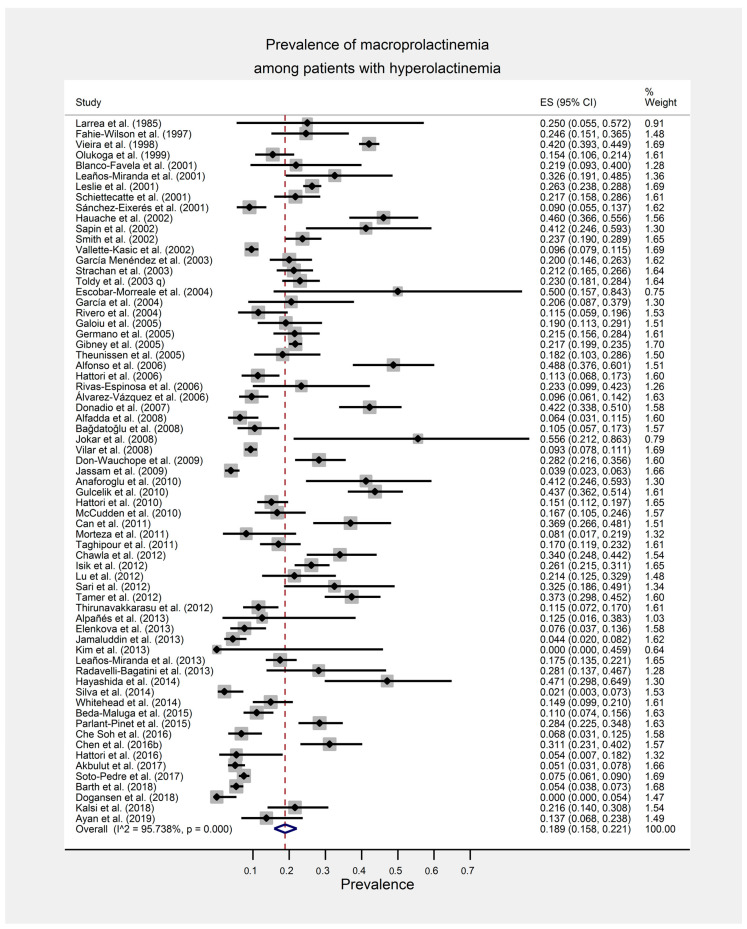
Forest plot of the meta-analysis for the global estimate of the prevalence of macroprolactinemia among patients with hyperprolactinemia.

Prevalence of mPRL among patients with hPRL from the included 67 studies ranged from 0.0% to 55.6% with a random effects pooled prevalence of 18.9% (95% CI: 15.8%, 22.1%) (Figure 2). There was a substantial statistical heterogeneity among the individual study estimates [χ^2^ (66) = 1548.67, *p* < 0.001, I^2^ = 95.7%].

### 3.3. Quality Assessment and Publication Bias

**Figure 3:**
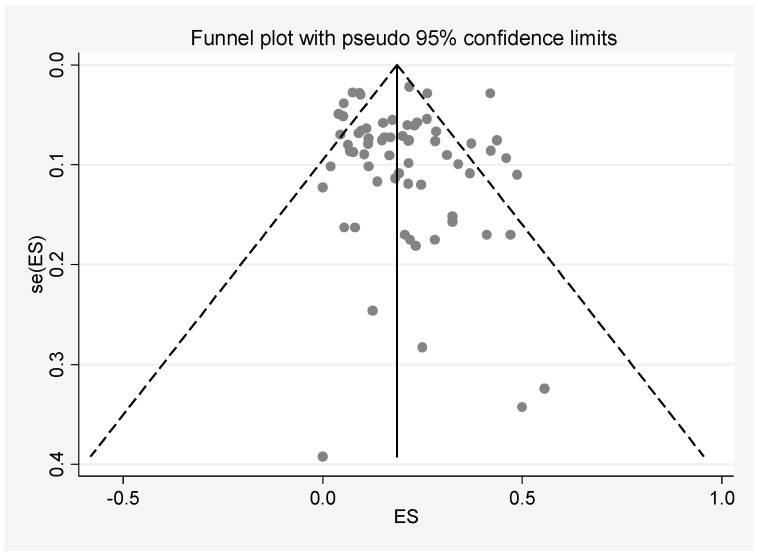
Funnel plot of publication bias. ES = Effect size estimate (prevalence).

Egger’s test for small-study effects indicates that there was no publication bias observed among all the included studies (*β* = 0.366; standard error of *β* = 0.418; 95% CI: −0.470, 1.201; *t* = 0.87, *p* = 0.385). The symmetry of the funnel plot agrees with the result of Egger’s test (Figure 3). A sensitivity analysis was conducted in which every study was removed in turn. The results showed no significant alterations in pooled prevalence and 95% CI values, indicating high stability of this meta-analysis (Supplementary Materials Table S1).

### 3.4. Subgroup and Meta-Regression Analyses

Variation in the prevalence estimate according to study region was explored by grouping the studies according to the World Health Organization Member States regions (African Region, Region of the Americas, South-East Asia Region, European Region, Eastern Mediterranean Region, and Western Pacific Region). The highest random effects pooled prevalence was observed in the African region (30.3%), followed by Region of the Americas (29.1%), European (17.5%), Eastern Mediterranean (13.9%), South-East Asian (12.7%), and Western Pacific Region (12.6%).

Further exploration of the variation in the prevalence estimate was made according to sex, age groups, year period of publication, and the types of PEG used for the detection of macroprolactin (PEG 6000 vs. PEG 8000). The summary of estimates and heterogeneity are summarized in Table 2.

For subgroup analysis of age group, eight studies were excluded because those studies did not specify the age group of their study participants. Age groups were classified as either involving adults only, teenagers only, or teenagers and adults. Year periods indicate the period of time when the study was published, categorized as either published before 2000, between 2000 and 2009, or between 2010 and 2019. For PEG type, the studies were categorized to either using PEG 6000 or PEG 8000. Studies that do not report the type of PEG used (*n* = 14) were excluded.

A statistically significant subgroup difference (interaction) was detected when the subgroup analysis was conducted according to sex (*p* = 0.010). A lower prevalence estimate was observed among studies involving both male and female participants as compared to studies involving only female participants (17.1% vs. 25.4%).

Meta-regression analysis reveals that the year of the studies had a significant influence on the prevalence where lower prevalence was observed in more recent studies (*p* = 0.010). No significant association was observed for sample size (*p* = 0.557) (Table 3).

## 4. Discussion

This meta-analysis showed an estimated prevalence of mPRL among patients with hPRL of 18.9%. Variation in the prevalence estimate was observed when the subgroup analysis was conducted according to the region. In the Region of the Americas and African Region, the subgroup analysis indicates a higher prevalence of mPRL, whereas in the European, Western Pacific, South-East Asian, and Eastern Mediterranean Region, the prevalence is slightly lower. The interpretation of this subgroup analysis, however, needs to be made with caution due to the small number of studies from the Western Pacific (*n* = 7), South-East Asian (*n* = 3), African (*n* = 2), and Eastern Mediterranean Region (*n* = 4). One study in the Region of the Americas reported a prevalence of 46%, and this finding reflected selection bias of the study because of the specialized nature of the study center. This center received samples from other laboratories when the possible diagnosis of mPRL was raised [33].

In this current meta-analysis, we could not compare the prevalence estimate between males and females as only one study was conducted with male participants [34]. Comparing studies conducted with female participants only to studies conducted with male and female participants reveals a significant difference. A lower prevalence estimate was observed among studies involving both sexes than studies involving only female participants. Findings from previous studies regarding the matter are inconclusive, with some studies reporting no difference in the prevalence of mPRL between sex [77,86,87,88]. In contrast, some other studies reported a higher prevalence of mPRL among females than males [35,36,51,89]. This could be due to a higher number of female patients being investigated for infertility and menstrual disturbance than men who are only being investigated for sexual dysfunction [69,90].

Subgroup analysis also did not show any difference in prevalence estimate based on age group, similar to other previous studies [49,88]. However, several other studies reported that the prevalence of mPRL tends to increase with advancing age [36,86].

Subgroup analysis by year periods reveals a reduction in the prevalence of mPRL in recent studies. Further evaluation by meta-regression analysis supports the finding and indicates that a lower prevalence estimate was reported in more recent studies. In this meta-analysis, however, we did not find any possible explanation for this variation.

A comparison between the type of PEG that has been used in the precipitation of macroprolactin shows a lower prevalence in studies that used PEG 8000 compared to those that used PEG 6000. However, only six studies used PEG 8000 as compared to 47 studies that used PEG 6000. A previous study reported a significant constant bias between the two macroprolactin precipitation methods. Therefore, they suggest laboratories that use PEG 8000 should consider the transference of the reference interval established with PEG 6000 carefully [91].

Among all studies included in this review, we found that various cut-offs for PRL level have been used for the screening of hPRL and mPRL with different percentages of PRL recovery post-PEG for diagnosis of mPRL. Immunoassays were performed using various systems, such as the Architect, DELFIA, Cobas, Elecsys, and IMMULITE. Variability of the PRL level based on the different immunoassay measurement system has been previously reported [32,79]. Apart from that, heterogeneity in the mPRL screening method between studies was observed. Some authors used only one method for either screening with PEG/ultrafiltration/protein A separation/protein G separation or GFC alone, whereas others chose to combine screening plus confirmation with GFC.

Several limitations need to be noted in this meta-analysis. Significant heterogeneity was identified, even though random effect models were carried out. This limitation is observed in any other meta-analyses of epidemiological studies, in which the source of heterogeneity may result from unreported factors. In this meta-analysis, we decided that it is important to show whether statistically significant subgroup differences exist based on subgroup analysis, even though there is considerable heterogeneity within subgroups. Between-group comparison based on the subgroup analysis, therefore, needs to be made with caution, and we acknowledge the uncertainty in the evidence due to inconsistency between individual study results.

In this meta-analysis, we could not examine the heterogeneity effect of sex (comparison between male and female) as only one study reported the prevalence of mPRL specifically among male hPRL patients. Similarly, the influence of age on the prevalence estimate could not be examined since only one study involved only teenagers, and no study involved only the elderly. All included studies were not race-specific, rendering the variation to be examined. Not all included studies report the exact protocol for sample collection, which may influence the level of PRL such as physiological stress and diurnal variation (serum PRL levels are known to be higher in the afternoon than in the morning). Furthermore, other common conditions that cause variability of PRL levels such as fasting state, exercise, history of drug intake, prior chest wall surgery or trauma, and comorbidities were not reported.

## 5. Conclusions

To the best of our knowledge, this is the first meta-analysis examining the global prevalence of mPRL among patients with hPRL. The pool prevalence of mPRL was 18.9% among patients with hPRL, indicating that the finding of mPRL is common among patients with hPRL. With macroprolactin causing nearly one-fifth of hPRL cases, screening for mPRL should be made routine before an investigation of other causes of hPRL.

## Figures and Tables

**Table 1:** Details of studies on the prevalence of macroprolactinemia among patients with hyperprolactinemia, sorted by year.

No	Author	Year	Country	Design	Age group	Sex	Specific Condition of hPRL	Method of PRL Detection	Method of Macroprolactin Detection	Cut off Recovery (%)	*n* hPRL	*n* mPRL
1	Larrea et al. [24]	1985	Mexico	cross-sectional	Adult	Female	No	RIA	GFC	-	12	3
2	Fahie-Wilson et al. [25]	1997	UK	cross-sectional	Adult	Both	No	FIA, EIA, CLIA	PEG 6000, GFC	-	69	17
3	Vieira et al. [26]	1998	Brazil	cross-sectional	Unspecified	Both	No	FIA	PEG 6000, GFC	30	1220	513
4	Olukoga et al. [27]	1999	UK	cross-sectional	Adult	Both	No	FIA	PEG 6000	40	188	29
5	Blanco-Favela et al. [28]	2001	Mexico	cross-sectional	Teenage	Both	SLE patients	IRMA	PEG, Protein G Sepharose	-	32	7
6	Leaños-Miranda et al. [29]	2001	Mexico	cross-sectional	Unspecified	Both	SLE patients	IRMA	PEG 6000, GFC	-	43	14
7	Sánchez-Eixerés et al. [30]	2001	Spain	cross-sectional	Adult	Both	No	ECLIA	PEG 6000, GFC	40	211	19
8	Schiettecatte et al. [19]	2001	Belgium	cross-sectional	Unspecified	Both	No	ECLIA	PEG 6000, GFC	50	175	38
9	Leslie et al. [31]	2001	UK	cross-sectional	Adult	Female	No	FIA	PEG 6000	40	1225	322
10	Smith et al. [32]	2002	UK	cross-sectional	Adult	Both	No	EIA, CLIA, ECLIA, IFMA	PEG, GFC	-	300	71
11	Hauache et al. [33]	2002	Brazil	cross-sectional	Adult	Both	No	FIA	PEG 6000, GFC	30	113	52
12	Sapin et al. [34]	2002	France	cross-sectional	All age	Male	No	CLIA, ECLIA	PEG 6000	40	34	14
13	Vallette-Kasic et al. [35]	2002	France	cross-sectional	All age	Both	No	CLIA	GFC	-	1106	106
14	Toldy et al. [36]	2003	Hungary	cross-sectional	All age	Both	No	ECLIA	PEG 6000	40	270	62
15	Strachan et al. [37]	2003	UK	cross-sectional	Adult	Both	No	CLIA	PEG 6000	50	273	58
16	García Menéndez et al. [38]	2003	Spain	cross-sectional	Adult	Both	No	ECLIA	PEG 6000	50	195	39
17	García et al. [39]	2004	Argentina	cross-sectional	Adult	Both	SLE patients	IRMA	PEG 6000, GFC	-	34	7
18	Escobar-Morreale et al. [40]	2004	USA	cross-sectional	Adult	Female	Hyperandrogenic	CLIA	PEG 6000	40	8	4
19	Rivero et al. [41]	2004	Spain	cross-sectional	Adult	Both	No	CLIA	PEG 6000, GFC	54	96	11
20	Galoiu et al. [42]	2005	Romania	cross-sectional	Adult	Both	No	IRMA, ECLIA	GFC, protein A precipitation	-	84	16
21	Germano et al. [43]	2005	Italy	cross-sectional	Adult	Both	No	CLIA	PEG 6000	40	172	37
22	Gibney et al. [18]	2005	Ireland	cross-sectional	Adult	Both	No	FIA	PEG 8000	-	2089	453
23	Theunissen et al. [44]	2005	Belgium	cross-sectional	Adult	Both	No	EIA, RIA, ECLIA	PEG 6000	40	77	14
24	Hattori et al. [45]	2006	Japan	cross-sectional	Teenage and adult	Both	No	ELISA	PEG 6000	40	159	18
25	Alfonso et al. [46]	2006	USA	cross-sectional	Adult	Both	No	ECLIA	PEG	50	82	40
26	Álvarez-Vázquez et al. [47]	2006	Spain	cross-sectional	Teenage and adult	Both	No	CLIA	PEG 6000	75	228	22
27	Rivas-Espinosa et al. [48]	2006	Mexico	others	Adult	Both	No	EIA	PEG 6000	50	30	7
28	Donadio et al. [49]	2007	Italy	retrospective cohort	Adult	Both	No	FIA	PEG 6000	40	135	57
29	Jokar et al. [50]	2008	Iran	cross-sectional	Teenage and adult	Both	SLE patients	RIA	PEG	40	9	5
30	Baǧdatoǧlu et al. [51]	2008	Turkey	cross-sectional	All age	Both	No	ECLIA	PEG 6000	40	124	13
31	Vilar et al. [52]	2008	Brazil	cross-sectional	Adult	Both	No	CLIA, IRMA	PEG	30	1234	115
32	Alfadda et al. [53]	2008	Saudi Arabia	retrospective cohort	All age	Both	No	ECLIA	PEG 6000	40	156	10
33	Jassam et al. [54]	2009	UK	cross-sectional	Adult	Both	No	CLIA	PEG 6000, GFC	40	409	16
34	Don-Wauchope et al. [55]	2009	South Africa	cross-sectional	All age	Both	No	CLIA	PEG 6000	60	170	48
35	Hattori et al. [9]	2010	Japan	cross-sectional	Adult	Both	No	EIA	PEG 6000	40	292	44
36	Anaforoglu et al. [56]	2010	Turkey	case-control	Adult	Female	No	CLIA	PEG 8000	40	34	14
37	McCudden et al. [11]	2010	USA	cross-sectional	Adult	Female	No	CLIA	PEG 6000	40	120	20
38	Gulcelik et al. [57]	2010	Turkey	cross-sectional	Adult	Both	No	CLIA	PEG	40	174	76
39	Taghipour et al. [58]	2011	Iran	cross-sectional	Adult	Both	No	ECLIA	PEG 6000	40	188	32
40	Can et al. [10]	2011	Turkey	cross-sectional	Adult	Female	No	CLIA	PEG 6000	40	84	31
41	Morteza et al. [59]	2011	Iran	longitudinal	Adult	Both	hPRL due to hypothalamus or stalk compression	IRMA	PEG	40	37	3
42	Thirunavakkarasu et al. [60]	2012	India	cross-sectional	Adult	Female	Infertility	ECLIA	PEG	40	183	21
43	Sari et al. [61]	2012	Turkey	cross-sectional	Adult	Both	Type 2 diabetes	ECLIA	PEG 8000	40	40	13
44	Isik et al. [62]	2012	Turkey	cross-sectional	Adult	Both	No	CLIA	PEG 6000	40	337	88
45	Tamer et al. [63]	2012	Turkey	cross-sectional	Adult	Female	No	ECLIA	PEG 6000	40	161	60
46	Chawla et al. [64]	2012	Ethiopia	cross-sectional	Adult	Female	No	ECLIA	PEG, GFC	40	100	34
47	Lu et al. [65]	2012	Taiwan	cross-sectional	Adult	Both	No	IRMA	PEG 6000	40	70	15
48	Kim et al. [66]	2013	Korea	cross-sectional	Adult	Both	Major depression on SSRI	CLIA	PEG 8000	52.8	6	0
49	Leaños-Miranda et al. [67]	2013	Mexico	cross-sectional	Adult	Female	Gynecological disorder	EIA	PEG 6000, GFC	-	326	57
50	Alpañés et al. [68]	2013	Spain	cross-sectional	Adult	Female	No	CLIA	PEG 6000	40	16	2
51	Radavelli-Bagatini et al. [69]	2013	Brazil	longitudinal	Adult	Female	No	IRMA	PEG 6000	40	32	9
52	Jamaluddin et al. [70]	2013	Malaysia	cross-sectional	Adult	Both	No	CLIA	PEG 6000, GFC	40	204	9
53	Elenkova et al. [71]	2013	Bulgaria	case-control	Adult	Both	Prolactinoma	RIA	PEG 8000	40	131	10
54	Whitehead et al. [72]	2014	Britain	cross-sectional	Unspecified	Both	No	CLIA	PEG 6000	-	175	26
55	Hayashida et al. [73]	2014	Brazil	cross-sectional	Adult	Female	PCOS	FIA	PEG 6000	30	34	16
56	Silva et al. [74]	2014	Portugal	cross-sectional	Unspecified	Both	No	ECLIA	PEG 6000	40	96	2
57	Beda-Maluga et al. [75]	2015	Poland	cross-sectional	Adult	Both	No	CLIA	PEG, Ultrafiltration, GFC	40	245	27
58	Parlant-Pinet et al. [76]	2015	France	cross-sectional	Adult	Both	No	RIA, ECLIA	PEG 6000, GFC	30	222	63
59	Che Soh et al. [77]	2016	Malaysia	cross-sectional	Adult	Both	No	ECLIA	PEG 8000	40	133	9
60	Chen et al. [78]	2016	China	cross-sectional	All age	Both	No	CLIA, ECLIA	PEG 6000, GFC	60	122	38
61	Hattori et al. [79]	2016	Japan	cross-sectional	Adult	Female	No	EIA, CLIA	PEG 6000, GFC	40	37	2
62	Akbulut et al. [80]	2017	Turkey	cross-sectional	Unspecified	Both	No	CLIA, ECLIA	PEG 6000	40	376	19
63	Soto-Pedre et al. [81]	2017	UK	longitudinal	Unspecified	Both	No	CLIA, ECLIA	unknown	-	1301	97
64	Dogansen et al. [82]	2018	Turkey	cross-sectional	Adult	Both	Prolactinomas	ECLIA	PEG 6000	40	66	0
65	Kalsi et al. [83]	2018	India	cross-sectional	Adult	Both	No	CLIA	PEG 6000	25	102	22
66	Barth et al. [84]	2018	UK	cross-sectional	Unspecified	Both	No	CLIA	PEG 6000	60	672	36
67	Ayan et al. [85]	2019	Turkey	cross-sectional	Adult	Both	No	ECLIA	PEG 6000	40	73	10

*n*: Number of patients with, RIA: Radioimmunoassay, FIA: Fluoroimmunoassay, CLIA: Chemiluminescence Immunoassay, ECLIA: Electrochemiluminescence Immunoassay, IRMA: Immunoradiometric Assay, IFMA: Immunofluorometric Assay, EIA: Enzyme Immunoassay, ELISA: Enzyme-Linked Immunosorbent Assay, PEG: Polyethylene glycol, GFC: Gel Filtration Chromatography, R: Recovery, SLE: Systemic lupus erythematosus, PCOS: Polycystic ovarian syndrome, hPRL: hyperprolactinemia, mPRL: macroprolactinemia, UK: United Kingdom, USA: United States of America.

**Table 2:** Subgroup analysis of the prevalence of macroprolactinemia among patients with hyperprolactinemia.

Study Characteristic	Number of Studies	Random Effect Pooled Prevalence	95% CI of Pooled Prevalence	Within Group Heterogeneity			Between Group Heterogeneity	
				I^2^ (%)	χ^2^ (df)	*p*-Value	χ^2^ (df)	*p*-Value
Region								
European Region	37	17.5	14.0, 21.2	95.7	840.70 (36)	<0.001	7.32 (3)	0.062
Region of the Americas	14	29.1	18.5, 41.0	97.1	455.07 (13)	<0.001		
Western Pacific Region	7	12.6	6.7, 19.9	89.3	55.94 (6)	<0.001		
South-East Asian Region	3	12.7	4.7, 23.1	-	-	-		
African Region	2	30.3	25.0, 36.0	-	-	-		
Eastern Mediterranean Region	4	13.9	4.8, 26.3	83.8	18.53 (3)	<0.001		
Sex								
Both (male and female)	52	17.1	13.8, 20.6	96.2	1359.49 (51)	<0.001	6.56 (1)	0.010
Female only	14	25.4	19.6, 31.6	84.9	86.49 (13)	<0.001		
Male only	1	41.2	24.6, 59.3	-	-	-		
Age group								
Adults only	48	19.8	16.6, 23.2	93.3	697.08 (47)	<0.001	0.23 (1)	0.630
Teenagers and adults	10	18.0	11.9, 25.0	92.2	114.91 (9)	<0.001		
Teenagers only	1	21.9	9.3, 40.0	-	-	-		
Year period								
Before 2000	4	26.5	11.2, 45.2	95.4	64.56 (3)	<0.001	2.64 (2)	0.267
Between 2000 and 2009	30	20.4	16.5, 24.5	94.6	536.29 (29)	<0.001		
Between 2010 and 2019	33	16.4	12.4, 20.9	94.3	557.34 (32)	<0.001		
PEG type								
PEG 6000	47	18.8	15.0, 23.0	95.6	1053.67 (46)	<0.001	0.06 (1)	0.801
PEG 8000	6	16.7	7.8, 27.7	90.6	53.43 (6)	<0.001		

PEG: Polyethylene glycol.

**Table 3:** Individual variable (univariable) meta-regression model for each study characteristic.

Study Characteristic	Number of Studies	Regression Coefficient (*β*)	Standard Error of *β*	95% CI of *β*	*t*	*p*-Value
Sample size	67	−0.00002	0.00003	−0.00008, 0.00004	−0.59	0.557
Year of the study	67	−0.007	0.003	−0.012, −0.002	−2.66	0.010

## Supplementary Materials

The following are available online at https://www.mdpi.com/1660-4601/17/21/8199/s1, Supplementary Table S1: “Leave-one-out” sensitivity analysis for the meta-analysis on the prevalence of macroprolactinemia among patients with hyperprolactinemia.
