# Supplementary material for: Global Prevalence of Macroprolactinemia among Patients with Hyperprolactinemia: A Systematic Review and Meta-Analysis
Source: Int J Environ Res Public Health. 2020 Nov 6;17(21):8199. doi: 10.3390/ijerph17218199 (PMC7664288; doi:10.3390/ijerph17218199)
Supplement: Supplementary file 1 [file ijerph-17-08199-s001.pdf]

Table S1. "Leave-one-out" sensitivity analysis for the meta-analysis on the prevalence of macroprolactinemia among patients with hyperprolactinemia.

Study Omitted	Prevalence Estimate (Coefficient) (%)	95% CI
Larrea et al. [24]	18.7	17.1, 20.2
Fahie-Wilson et al. [25]	18.6	17.1, 20.1
Vieira et al. [26]	16.8	15.3, 18.4
Olukoga et al. [27]	18.7	17.2, 20.2
Blanco-Favela et al. [28]	18.7	17.1, 20.2
Leaños-Miranda et al. [29]	18.6	17.1, 20.1
Sánchez-Eixerés et al. [30]	18.8	17.3, 20.3
Schiettecatte et al. [19]	18.6	17.1, 20.1
Leslie et al. [31]	18.1	16.5, 19.6
Smith et al. [32]	18.6	17.1, 20.1
Hauache et al. [33]	18.5	17.0, 20.0
Sapin et al. [34]	18.6	17.1, 20.1
Vallette-Kasic et al. [35]	19.3	17.7, 20.8
Toldy et al. [36]	18.6	17.1, 20.1
Strachan et al. [37]	18.6	17.1, 20.1
García Menéndez et al. [38]	18.6	17.1, 20.2
García et al. [39]	18.7	17.1, 20.2
Escobar-Morreale et al. [40]	18.6	17.1, 20.1
Rivero et al. [41]	18.7	17.2, 20.2
Galoiu et al. [42]	18.7	17.1, 20.2
Germano et al. [43]	18.6	17.1, 20.1
Gibney et al. [18]	18.2	16.6, 19.8
Theunissen et al. [44]	18.7	17.2, 20.2
Hattori et al. [45]	18.7	17.2, 20.2
Alfonso et al. [46]	18.5	17.0, 20.0
Álvarez-Vázquez et al. [47]	18.8	17.3, 20.3
Rivas-Espinosa et al. [48]	18.7	17.1, 20.2
Donadio et al. [49]	18.5	17.0, 20.0
Jokar et al. [50]	18.6	17.1, 20.1
Bağdatoğlu et al. [51]	18.7	17.2, 20.2
Vilar et al. [52]	19.4	17.8, 21.0
Alfadda et al. [53]	18.8	17.3, 20.3
Jassam et al. [54]	19.0	17.5, 20.5
Don-Wauchope et al. [55]	18.6	17.1, 20.1
Hattori et al. [9]	18.7	17.2, 20.2
Anaforoglu et al. [56]	18.6	17.1, 20.1
McCudden et al. [11]	18.7	17.2, 20.2
Gulcelik et al. [57]	18.4	16.9, 19.9
Taghipour et al. [58]	18.7	17.2, 20.2
Can et al. [10]	18.6	17.1, 20.1
Morteza et al. [59]	18.7	17.2, 20.2
Thirunavakkarasu et al. [60]	18.7	17.2, 20.2
Sari et al. [61]	18.6	17.1, 20.1
Isik et al. [62]	18.5	17.0, 20.0
Tamer et al. [63]	18.5	17.0, 20.0
Chawla et al. [64]	18.6	17.1, 20.1
Lu et al. [65]	18.6	17.1, 20.2
Kim et al. [66]	18.7	17.2, 20.2

Leaños-Miranda et al. [67]	18.7	17.2, 20.2
Alpañés et al. [68]	18.7	17.2, 20.2
Radavelli-Bagatini et al. [69]	18.6	17.1, 20.1
Jamaluddin et al. [70]	18.8	17.3, 20.3
Elenkova et al. [71]	18.7	17.2, 20.3
Whitehead et al. [72]	18.7	17.2, 20.2
Hayashida et al. [73]	18.6	17.1, 20.1
Silva et al. [74]	18.8	17.2, 20.3
Beda-Maluga et al. [75]	18.8	17.3, 20.3
Parlant-Pinet et al. [76]	18.5	17.0, 20.0
Che Soh et al. [77]	18.8	17.2, 20.3
Chen et al. [78]	18.6	17.1, 20.1
Hattori et al. [79]	18.7	17.2, 20.2
Akbulut et al. [80]	19.0	17.4, 20.5
Soto-Pedre et al. [81]	19.6	18.0, 21.2
Dogansen et al. [82]	18.7	17.2, 20.2
Kalsi et al. [83]	18.6	17.1, 20.1
Barth et al. [84]	19.2	17.7, 20.7
Ayan et al. [85]	18.7	17.2, 20.2
